# Deconstructing Ecosystem Service Conflicts through the Prisms of Political Ecology and Game Theory in a North-Western Mediterranean River Basin

**DOI:** 10.1007/s10745-022-00325-5

**Published:** 2022-05-26

**Authors:** Enrica Garau, Josep Pueyo-Ros, Josep Vila-Subiros, Anna Ribas Palom

**Affiliations:** 1 Department of Geography, Institute of Environment, IMA-UdG, University of Girona, 17071 Girona, Spain; 2 ICRA, Catalan Institute for Water Research, 17003 Girona, Spain

**Keywords:** Socioecological systems, Water ecosystem services, Stakeholder power relationships, River basin management, Muga river basin, Northeast Catalonia

## Abstract

Power relationships, access and control, (in)equity, and (in)justice are key modulators of conflicts arising from ecosystem services between multiple stakeholders. A greater knowledge of stakeholder value systems and behaviors is crucial for understanding socioecological dynamics. We propose an analytical framework that combines political ecology and game theory to analyze water ecosystem services. This integrated framework was used to reinterpret concepts such as common goods, (a)symmetric flows, and (un)fair trade-offs in the context of ecosystem services. The purpose was to gain a better understanding of behaviors and (a)symmetries in power relationships between multiple stakeholders. We studied the case of a north-western Mediterranean river basin using data obtained from stakeholder interviews and newspaper articles. Our findings uncovered different types of stakeholder relationships, ranging from mutual support and cooperation to conflict. The proposed analytical framework shows how social mechanisms can affect ecosystem services flows, potentially facilitating or hindering the development of more equitable management models for natural resources.

**Supplementary Information:**

The online version contains supplementary material available at 10.1007/s10745-022-00325-5.

## Introduction

Water ecosystems and their service flows have been dramatically altered by human activity throughout history, making them one of the most threatened ecosystems in the world (Dudgeon et al., 2006; Green et al., 2015; Podimata & Yannopoulos, 2015) and placing them at the center of the debate on water scarcity and social conflict. They are a key priority on political agendas across the globe (Green et al., 2015; Grizzetti et al., 2016; Vollmer et al., 2018).

Water ecosystem service (WES) flows, understood as the connection between WES supply and demand areas-units (Palomo et al., 2013), are not always evenly distributed in terms of space (Green et al., 2015) or access (Felipe-Lucia et al., 2015). The benefits offered by these flows are multiple and include water for human consumption and production purposes (provisioning services); water quality, climate and water regulation, and habitat and biodiversity conservation (regulating services); and recreational activities and aesthetic and symbolic values (cultural services). They depend not only on the existence of provisioning hotspots but also on sociocultural factors, acceptance of management policies among stakeholders, power relationships, control and access structures, and demand and needs (Castro et al., 2013; Quintas-Soriano et al., 2014). These social mechanisms are also linked to the ecological status of the ecosystem and the intensity of its flows, that is the quantity, distribution, and availability of services (Felipe-Lucia et al., 2015; Kretsch & Kelemen, 2016; Palomo et al., 2016). Water ecosystems can thus clearly be perceived as dynamic socioecological systems (Vollmer et al., 2018).

Numerous studies have analyzed ES flows using a range of approaches, including supply and demand hotspots, trade-offs and bundles (synergies), and the spatial distribution of beneficiaries (e.g., Burkhard et al., 2014; García-Nieto et al., 2013; Iniesta-Arandia et al., 2014; Jacobs et al., 2015; Palomo et al., 2013; Quintas-Soriano et al., 2014; Zoderer et al., 2019). Few studies, however, have examined the power relationships between multiple ES beneficiaries, and they have all considered demand from the perspective of single beneficiaries, failing to make “explicit reference to different groups of humans who unevenly share the different benefits and costs of ES”, as stated by Daw et al. (2011: 370). Aggregated approaches can be problematic as they look at society as a whole (Rincón-Ruiz et al., 2019). An analysis, for example, of the benefits of forest ES to human well-being without consideration of the value of these services to farmers, foresters, or logging companies will miss important differences in value systems, management decisions, and the distribution of benefits. A disaggregated analysis, by contrast, with consideration of individual stakeholder groups, may provide more accurate information (Daw et al., 2011).

Several studies have analyzed ES through the theoretical framework of political ecology and the concepts of social and environmental justice, with a particular focus on the distribution of trade-offs and their impact on beneficiaries and ES (Fisher et al., 2013, 2014; Luck et al., 2012). Environmental justice is understood as the environmental advantages or disadvantages that different social groups can experience in relation to equal access and protection of environmental laws and regulations relating to a spatial scale (Mohai et al., 2009). These studies criticize a conceptual approach to ES grounded in a purely ecological dimension that largely ignores the political connotations of ES that can exacerbate existing situations of social injustice and unequal power relationships (Daw et al., 2011). Stakeholders in an upper river basin, for example, may benefit from better water quality than those located downstream, who are strongly dependent on the actions of their upriver counterparts. The existence of certain stakeholders with a better understanding of how and when ES management decisions are taken or with a greater power to influence these decisions can lead to short- and long-term environmental injustices, posing a major challenge in this field (Kretsch & Kelemen, 2016; Rincón-Ruiz et al., 2019).

The aim of this study was to narrow the knowledge gap in the field of ES conflicts and injustices related to access, control, and distribution of WES among beneficiaries by disentangling these frictions in a Mediterranean river basin through the prism of two complementary theoretical frameworks: political ecology and game theory. To do this, we explored control, access, and power dynamics among multiple stakeholders competing for WES in the basin based on a reinterpretation of several concepts such as common goods, asymmetry of ES flows, and trade-off fairness. We illustrated our proposal by applying three research questions to a case study of the Muga river basin in the north-western Mediterranean: a) Do different stakeholder groups have equitable access and entitlements to WES benefits in the study area? b) Are WES flows affected by (a)symmetry in power relationships (control and access) between the different stakeholders? and c) Whose interests do decisions that affect and modify WES flows serve?

## Analytical Framework: Conceptualizing WES Conflicts through the Prisms of Political Ecology and Game Theory

The analytical approach in this study combines the theoretical frameworks of game theory and political ecology to study potential behaviors and conflicts that can arise from water resource management among stakeholders considered as players in a game involving aspects linked to power relationships, access and control, equity, and environmental justice (Felipe-Lucia et al., 2015). We chose game theory and political ecology as they have been widely used to study conflicts and social tensions surrounding the use of natural resources, including water (Dinar & Hogarth, 2015) and their combined use can provide insights into stakeholder behaviors and help identify strategic solutions to water-related problems.

**Fig. 1:**
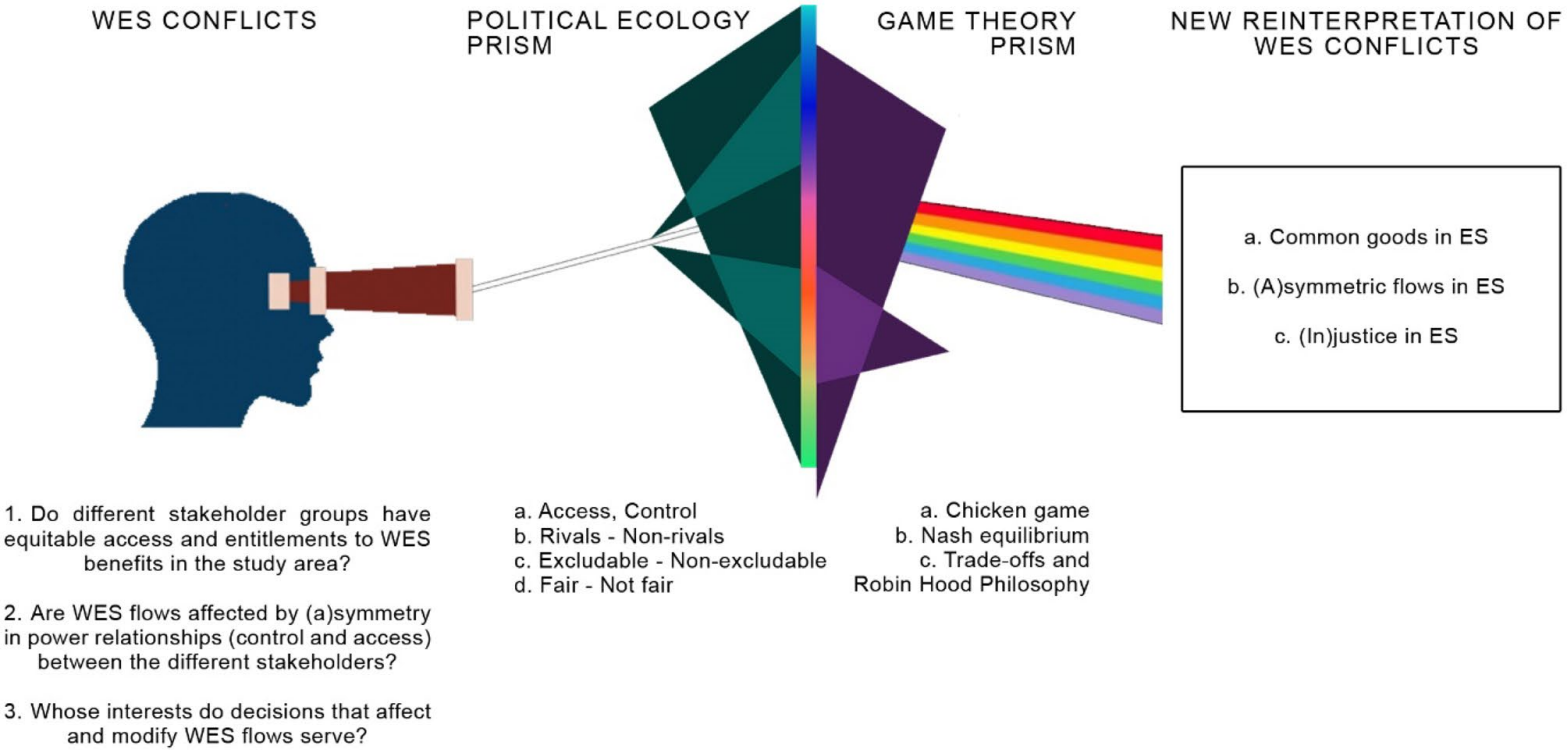
Construction of the analytical framework through the prisms of political ecology and game theory

We built our analytical framework by 1) defining empty signifiers, which are key universal principles used in political ecology that acquire different meanings depending on when and where they are used (rivalry, excludability, justice, and equity) (Popartan et al., 2020) and 2) choosing the game theory models to apply at the conceptual level. The integration of the two frameworks provided us with analytical tools filled with meaning (particular signifiers) to deconstruct WES conflicts into their different dimensions. The steps followed are shown in Fig. 1 and the details of the terms and combined analytical frameworks are explained in subsections "Adjustment to Political Ecology Principles: Converting Empty Signifiers to Particular Signifiers in the Framework of ES" and "Adjustment to Game Theory Principles: Selection and Application of Game Theory Tools to ES" of the Analytical Framework section.

### Adjustment to Political Ecology Principles: Converting Empty Signifiers to Particular Signifiers in the Framework of ES

Political ecology helps us understand how global discourses and material phenomena unfold in local contexts (Adger et al., 2000; Fisher et al., 2013, 2014; Rocheleau, 2008), providing key insights into the complexity of local realities (Barnaud & Antona, 2014). To analyze conflicts surrounding WES, it is first necessary to define what is meant by a common good and determine what is understood by fair/unfair and equitable/inequitable and by whom. To do this, we adapted the definitions of some of the key principles of political ecology (common goods, tragedy of the commons, power relationships, conflicts, and fair/unfair trade-offs) to our case study by converting these empty signifiers into WES-related signifiers (see Box 1).

Box 1. Access, control, rivalry, and excludability. Water ecosystem services (WES): Common goods or tragedy of the commons? Common goods. Common goods in economics are goods considered to be rivalrous and non-excludable (Hardin, 1968a, b). As such, they meet two requisites: rivalry, when the consumption of a good by one person precludes its consumption by another, and excludability, when it is not possible to prevent a person who has not paid for a good from accessing it (Ostrom, 1990).

Tragedy of the commons. The tragedy of the commons is a situation in a shared-resource system where individual users, acting independently according to their own self-interest, behave contrary to the common good of all users by depleting or spoiling the shared resource through their collective action (Hardin, 1968a, b; Lloyd, 1883; Ostrom, 1999).

Conflict. A conflict is a situation in which two or more decision-makers enter into a dispute over a given issue (Raquel et al., 2007).

WES as common goods (free access–non-excludability). In the absence of excludability mechanisms and in a scenario of free access to WES flows by all stakeholders (non-excludability), WES can be considered to be common goods (Kretsch et al., 2016).

The tragedy of WES (controlled access – excludability). When a stakeholder has a strong level of influence on decision-making and control of and access to WES flows, these services can be considered to represent a tragedy of the commons (Lant et al., 2008).

Congestible WES. A WES that can change from a non-rival good to a rival, excludable good, depending on its ecological status and relationship with stakeholders who benefit from it (Felipe-Lucia et al., 2015).

*WES power relationships and (a)symmetries.*

Power relationship. A relationship in which one person has the ability to control or influence another person’s access to WES (Felipe-Lucia et al., 2015).

Symmetric power relationship. Situation in which stakeholders employ a strategy designed to achieve optimal distribution of WES flows, benefiting the maximum number of stakeholders (win-win situation) and in the absence of excludability mechanisms (equitable distribution of WES) (Felipe-Lucia et al., 2015);

Asymmetric power relation. Situation in which stakeholders act in a non-cooperative manner, prioritizing individual over collective gains in the presence of excludability mechanisms (win-lose situation) and leading to the creation of trade-offs between WES (inequitable distribution) (Felipe-Lucia et al., 2015).

Trade-off. Situation in which land use or management actions by a stakeholder or group of stakeholders increase the provision of a WES to the detriment of another. This may be due to simultaneous responses to the same driver or true interactions between services. Adapted from (Bennett et al., 2009).

*Fair – Unfair. The Robin Hood Philosophy*

The Robin Hood philosophy. The Robin Hood philosophy is based on stealing from the rich to give to the poor with the aim of achieving a fairer distribution of “goods” or resources among everyone.

Fair trade-offs. Trade-offs that follow the Robin Hood philosophy and benefit as many stakeholders or groups of stakeholders as possible while creating a fairer distribution of resources overall.

Unfair trade-offs. Trade-offs that favor single stakeholders or groups of stakeholders, negatively affecting the flow of WES to others and creating an unfair distribution of resources.

### Adjustment to Game Theory Principles: Selection and Application of Game Theory Tools to ES

Game theory is a well-known methodological framework used to study conflicts and cooperation behaviors through mathematical modelling. Game theory models serve to predict the behavior of different agents based on the logical and strategic study of their decisions (Myerson, 1991). These decisions respond to individual interests and gains that each agent wants to maximize (Madani, 2010; Zanjanian et al., 2018). Game theory has been widely applied to study conflicts surrounding the management of natural resources, such as forest management (Rodrigues et al., 2009), water governance (Sullivan et al., 2019), and zoning of protected areas (Lin & Li, 2016). Hypothetical solutions or contexts must have three basic ingredients to qualify as a game theory model (Gibbons, 1997; Najafi et al., 2013): i) agents, ii) value systems and actions, and iii) payoffs. Agents are stakeholders who act of their own free will, making decisions independently or in collaboration with others. All stakeholders have a value system and take decisions in pursuit of individual or collective gain. Understanding this value system is key to understanding what each stakeholder values (or does not value), what their priorities are, and how they act as a result. To an extent, a person’s value system can be viewed as the driving force behind their decisions and choices. Finally, payoffs are the gains or losses (money, utility, personal gain) that stakeholders receive when they act in a certain way.

Let us assume that the game setting is a river basin with different agents interacting and making decisions (stakeholders). Each agent wants to maximize their gains, and this may or may not negatively impact the gains that other stakeholders can derive from water ecosystems and the services they produce (Rodrigues et al., 2009). Payoffs are the sum of all gains, as gains to one party can be affected by decisions or actions taken by others. Although in game theory it is assumed that players are rational agents who act to maximize their outcomes, their choices may also be influenced by mechanisms such as pressure, risk aversion, or power relationships (Podimata & Yannopoulos, 2015).

Under the above assumptions, we applied several game theory models to WES conflicts in the Muga river basin to explore strategies and actions taken by stakeholder groups with different power relationships and ability to access and control WES flows (Fisher et al., 2014).

To analyze the conflicts, we applied the Chicken game model and the Nash equilibrium (Najafi et al., 2013). Chicken is a game with winners and losers. It is represented by two players driving towards each other along a narrow road. They can choose to swerve and avoid hitting the other car (in which case they are labeled a chicken) or to not swerve and keep going (in which case they are labeled a hero). If they both decide to keep going, they will crash, which is the worst possible outcome or payoff (lose-lose situation); if they both decide to swerve, they will have to deviate from their original course, but they will both survive (suboptimal win–win situation); if one decides to swerve and the other decides to keep going, one will lose and the other will win (win-lose situation). The players’ choices will be guided not by the harm they think they might cause the other but by what they believe is best for them.

The Nash equilibrium is a solution criterion used in game theory. It is the situation where the sum of payoffs is the best possible outcome for all players (Eleftheriadou & Mylopoulos, 2008). We used the Nash equilibrium point and the Robin Hood philosophy to classify trade-offs or bundles between WES as fair or unfair, depending on whether they benefited the maximum number of stakeholders and resulted in an equitable or an inequitable distribution of WES.

**Fig. 2:**
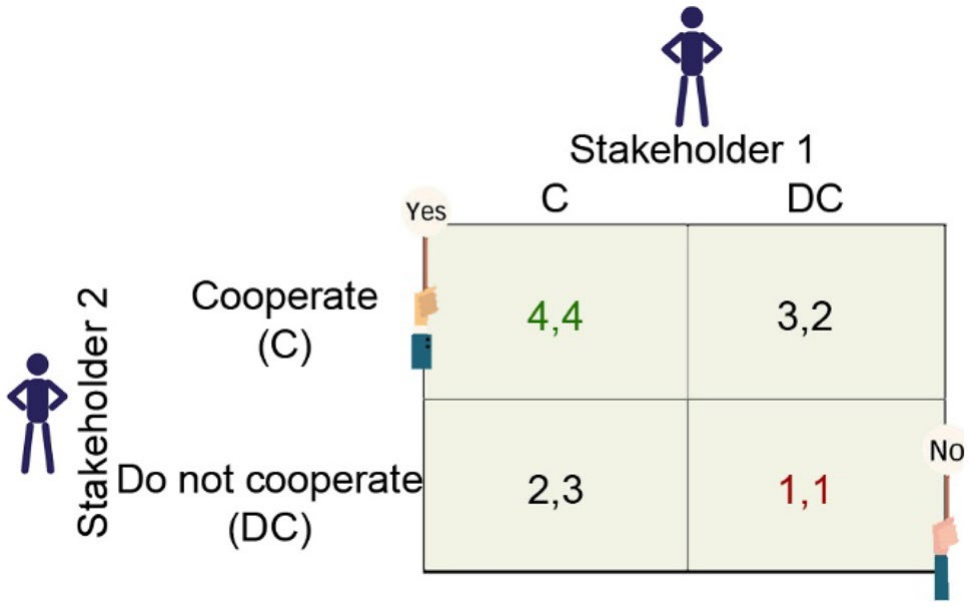
Theoretical representation of a game simulation applied to water ecosystem services (WES). The numbers represent the sum of gains that stakeholders #1 and #2 will derive from the WES depending on the strategy they choose (cooperate [C] vs. do not cooperate [DC]). Higher numbers represent more gains and a more sustainable management of WES flows

These theoretical models are shown as 2 × 2 games in Fig. 2 (Madani, 2010), where the numbers, derived from theory, represent the payoffs resulting from the strategy chosen by each stakeholder in relation to the other.

Figure 2 specifically represents the conceptual model of a harmony game, where there are no conflicts, and the agents choose to cooperate with each other. As shown, a cooperation strategy [C–C] maximizes the sum of gains for both stakeholders (WES = 4). On the contrary, the worst possible strategy is for neither of the stakeholders to cooperate, as this will result in the greatest losses to both parties (WES = 1) and degrade the ecological status of water ecosystems.

**Fig. 3:**
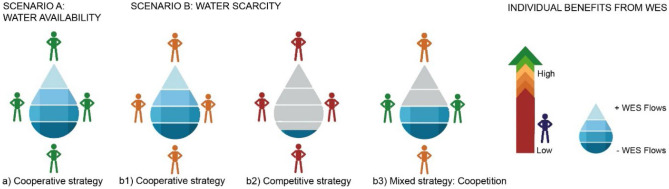
Stakeholder strategies, benefits, and distribution of water ecosystem service (WES) flows in a context of water availability (**A**) (harmony game) and water scarcity (**B**) (chicken game). The fuller the drop of water, the more benefits for all involved and better the management of WES flows

In addition, depending on the socioecological context (plentiful vs scarce resources), the stakeholders can choose to engage in cooperative or non-cooperative behavior, which could alter their relationship (causing a shift from a positive-supportive to a negative-conflictive relationship) and the flows of WES. Figure 3 shows the benefits that stakeholders would receive in different scenarios.

## Application of the Analytical Framework to a North-Western Mediterranean River Basin

### Case Study Description

**Fig. 4:**
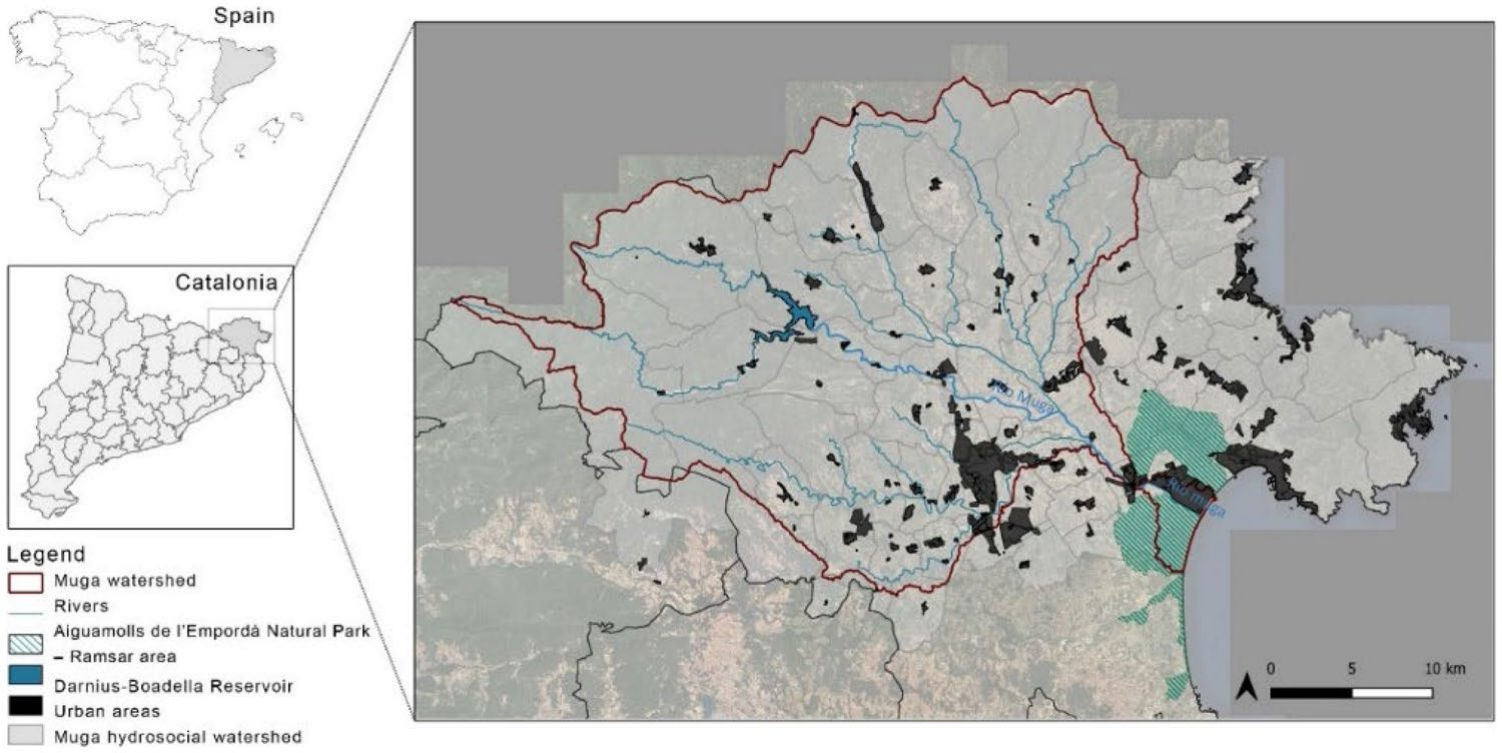
Muga river basin

The study area is the Muga river basin, located in northeast Catalonia on the border between France and Spain (Fig. 4).

**Fig. 5:**
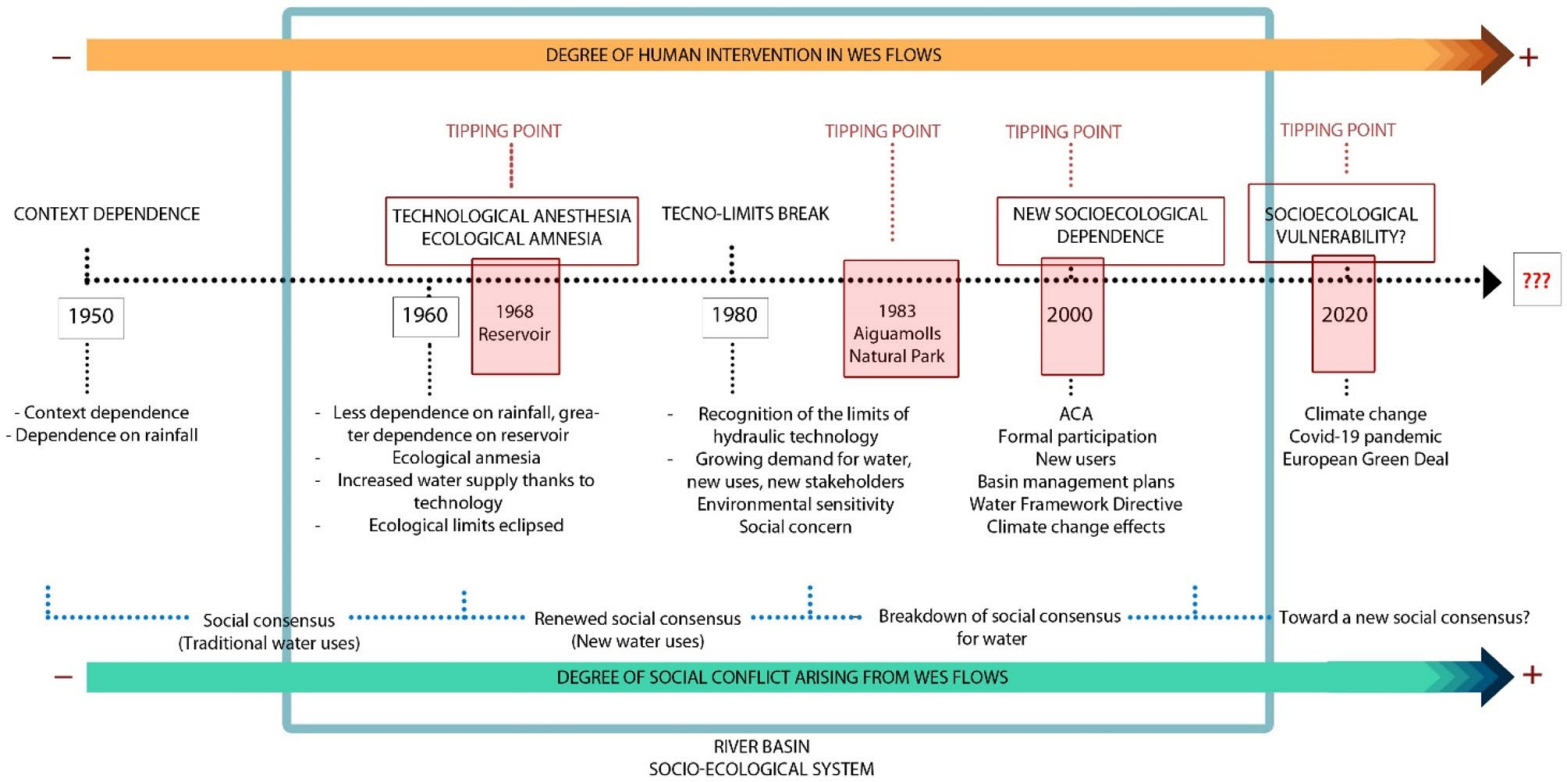
Key tipping points in relation to water resources in the Muga river basin from 1950 to 2020. The acronym ACA means Catalan Water Agency

The Muga river runs for 64 km through a basin with a surface area of 854 km^2^. It rises in the Pre-Pyrenees at an altitude of 1200 m and flows into the Gulf of Roses through the marina in Empuriabrava. With a mean annual flow of 2.5 m^3^/s (IDESCAT, 2020), the river has a typically Mediterranean regime, although its flow is regulated by the Darnius-Boadella reservoir, the main source of water for the basin. Since the mid-twentieth century, the basin has experienced a progressive increase in intensive crop and livestock farming and urban and tourism development, particularly along the coast. The particularities of the basin, coupled with changing trends in recent decades, have increased the demand for increasingly scarce water supplies, fueling both tensions and conflicts (Saurí et al., 2000; Tàbara et al., 2004). The Muga river basin is divided into three main areas: the headwaters (upper basin), consisting mainly of mountains and forestland and featuring the Darnius-Boadella reservoir to the south; a central area (the middle basin), home to one of Catalonia’s largest agricultural plains and the capital of the region, Figueres; and a coastal area (the lower basin), a renowned international tourist resort (Gabarda-Mallorquí & Ribas, 2016; Torres-Bagur et al., 2019) and home to the Aiguamolls de l'Empordà Natural Park (Category V – IUCN), a protected natural area that has been a member of the Ramsar International Network of Protected Wetlands since 1993 (Ramsar, 1999). The Muga river basin is thus an extraordinarily diverse area in terms of ecosystems, landscapes, and socioeconomic activity. This extraordinary diversity has led to social changes, conflicts surrounding water quality, quantity, and distribution, and socioecological tipping (inflection) points (Bentley et al., 2014) related to the use of water resources (see Fig. 5).

### Data Collection

The stakeholders to be interviewed were selected by non-proportional quota sampling, since the purpose was to ensure that all groups, both large and small, were adequately represented (Raymond et al., 2009; Tashakkori & Teddlie, 2003). Thirty-two stakeholders were contacted and 27 agreed to participate (Table 1). Separate interviews were held with each of these stakeholders, who were from different sectors directly or indirectly involved in WES (academia, agriculture, recreational on-site tourism, conservation, government – technical level, government – political level). Two hydroelectric power companies and three hotels refused to participate.

**Table 1:** Stakeholders interviewed

Sector	No. of stakeholders interviewed
Research	1 + 6 (expert panel)
Agriculture	4
Recreational on-site tourism	6
Tourism business sector	4
Conservation	5
Government – technical level	4
Government – political level	2

The field work was carried out between June and November 2019. The interview was structured into five parts, each addressing a different topic: (i) familiarity with the study area, (ii) familiarity with the concept of ES, (iii) perceptions of WES importance, demand, vulnerability, and spatial position, (iv) perceived problems (existing and future) and concerns about water resources, and (v) socioeconomic profile (gender, age, place of residence, etc.) (Supplementary information A). The interview model contained open and closed questions to give the interviewees the opportunity to express their opinions freely and to explore given topics in greater depth (Iniesta-Arandia et al., 2014). All the interviews were audio-recorded and transcribed in full. The transcripts were analyzed and coded into categories in Maxqda (v. 10, 2012) and the quantitative analysis was performed in Jamovi (v. 1.0.7.0). Discourse analysis was applied to analyze the content of the transcripts (Hatton MacDonald et al., 2013).

The interview content analysis was complemented by a systematic search of local and regional newspaper archives published between January 1, 2000, and July 31, 2020 using the keywords “water” and “Alt Empordà”. The year 2000 was chosen as the starting point as it was identified as a key “social tipping point” within the framework of a new socioecological dependence linked to the adoption of the European Union Water Framework Directive. It was also when the more serious effects of climate change were starting to become evident (see Fig. 5). Although we are aware that news can sometimes be distorted to attract or diverting media attention to certain topics (McLellan & Shackleton, 2019), we considered it to be an important source of information as newspapers are a useful proxy for issues of concern to society at a given time (Clegg Smith et al., 2002; Lawhon & Makina, 2017). To reduce potential bias, we chose a diverse range of local and regional newspapers with different editorial lines: *La Vanguardia, Ara.cat, El Punt Avui*, *Diari Girona* (daily newspapers) and *Hora Nova* and *Empordà* (weekly newspapers).

The search retrieved 2386 news items, of which 147 met the selection criteria: date of publication (2000–2020), place (Muga river basin), and topic (water conflict, management, or uses, water ecosystems, and WES).

All the news items selected were classified using an inductive coding process to characterize conflicts, stakeholders, WES, dominant relationships, and trade-offs or bundles (Supplementary information D). The content of the interviews, conducted in 2019, has been analyzed in depth in a previous study (Garau et al., 2020). In the current study, we collated the interview findings with the data from the newspaper analysis, corresponding to the period 2000–2020.

### WES Conflicts in the Basin

The 27 stakeholders were divided into four groups of agents: the agricultural sector (crop and livestock farmers), the conservationist sector (managers of natural protected areas, environmental groups, and environmentalists), the urban-tourism sector (urban actors, tourism businesses, and recreational businesses), and the government sector (decision makers at different levels of government and regulators of water resource management decisions). We combined the urban and tourism sectors as the news analysis showed they had very similar interests, especially regarding the distribution and quality of water for human consumption.

**Fig. 6:**
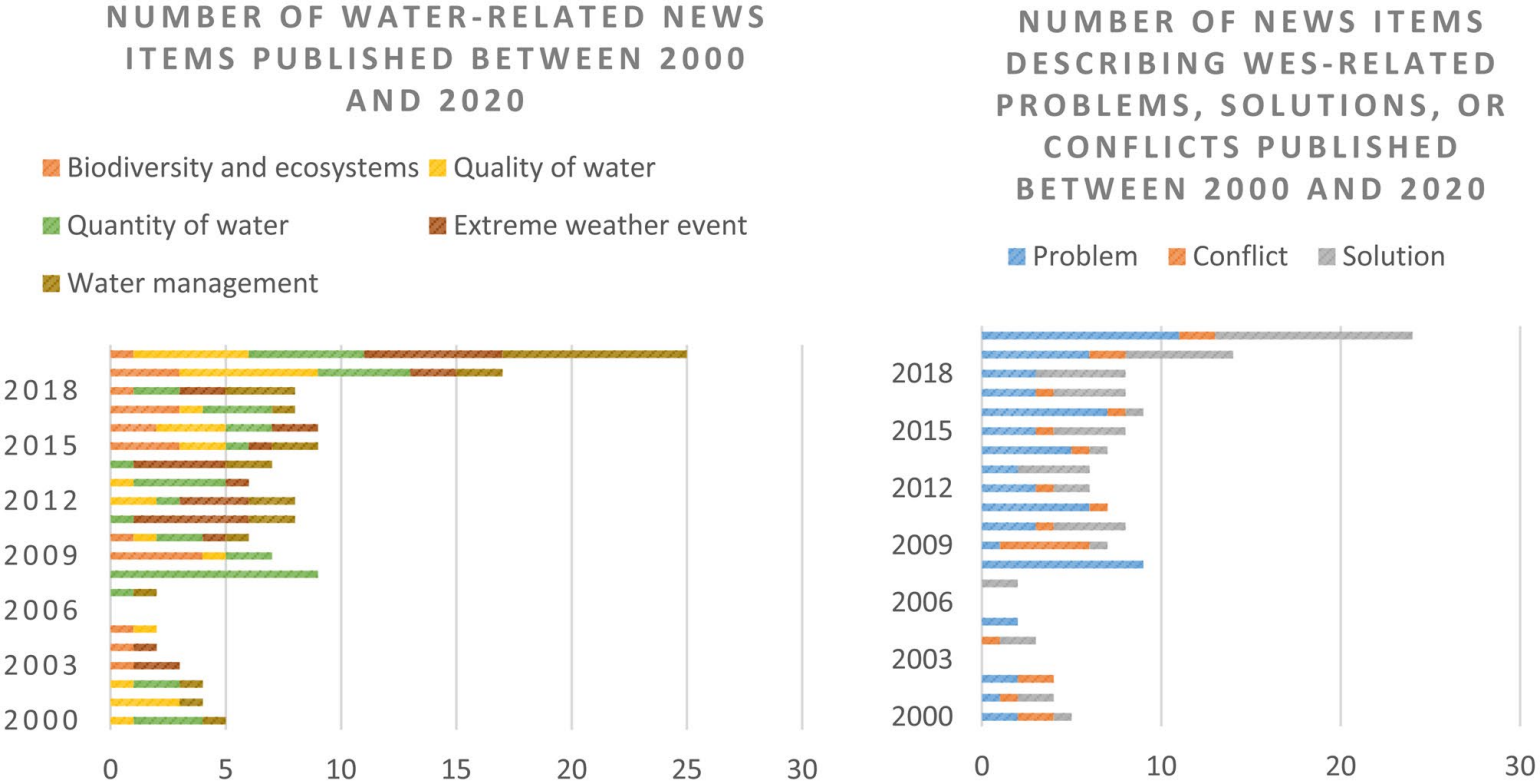
Number of news items on water resources in the Muga river basin shown by theme (**A**) and type of news (**B**)

The most common themes covered in the 147 news items were quantity of water (27.9%), extreme weather events (21.1%), water quality (19%), resource management (administrative/economic) (18.4%), and biodiversity and ecosystem management (13.6%). The distribution of these items is shown in Fig. 6. The peak observed in 2020 reflects the increasing socioecological dynamics emerging in the river basin in relation to both the effects of climate change on the distribution of WES flows (quality and quantity of water and extreme weather events) and the increasing difficulty of managing new water demands and uses (Fig. 6A). At the same time, and similarly to other authors (Holt & Barkemeyer, 2012; McLellan & Shackleton, 2019), we detected a clear increase in the coverage of WES problems and solutions (Fig. 6B) in 2007; coverage remained fairly consistent over the next decade and peaked in 2018, coinciding with a prolonged period of extreme drought and water shortages in the Darnius-Boadella reservoir. Coverage increased again in 2019 and 2020, with growing reports on extreme weather events and climate change, groundwater contamination, well salinization, and, in a context of increasing water scarcity, restrictions on water use in various sectors.

**Fig. 7:**
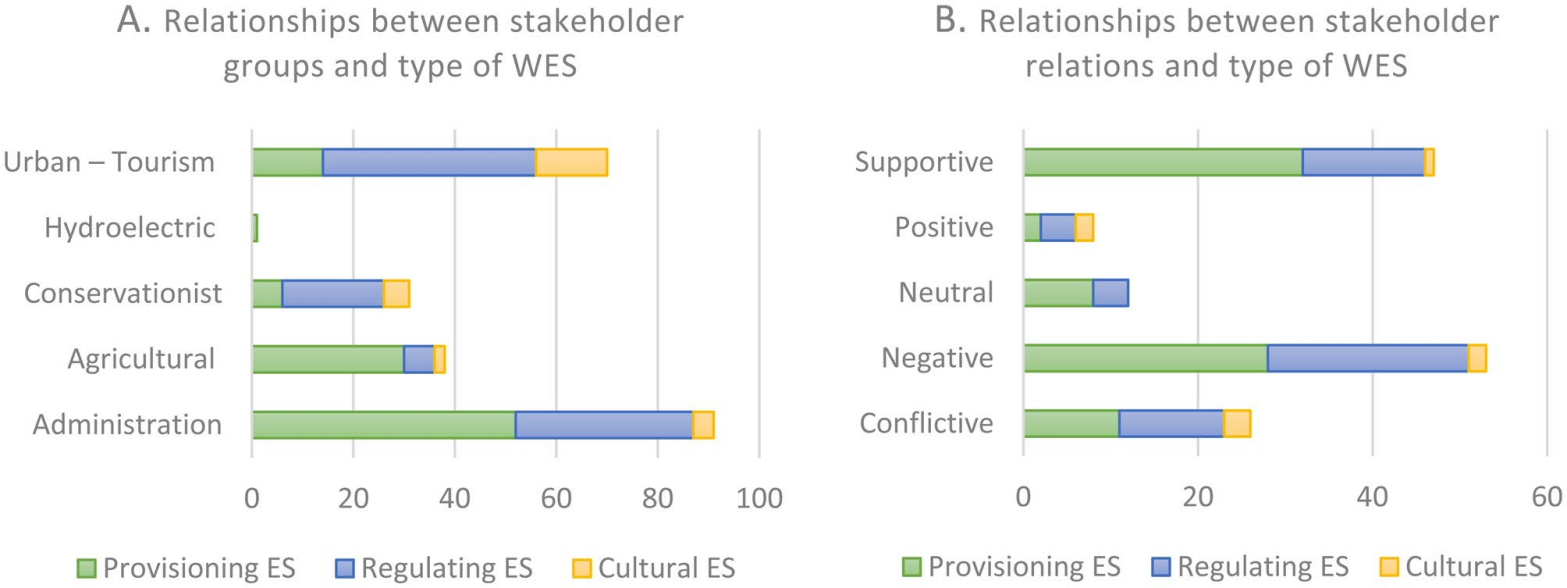
Relationships between stakeholder groups and WES reported in the news shown by type of WES (**A**) and type of relationship (**B**)

The conservationist sector was mentioned more in conflicts related to regulating WES (water regulation, quality, erosion control, biodiversity conservation, and ecosystem management), while the agricultural and urban-tourism sectors were more involved in conflicts related to provisioning WES (water for domestic use or irrigation and food production) (Fig. 7A). Relationships between stakeholders and WES were depicted as positive/supportive in 41.5% of the news items and as negative/conflictive in 58.5% (Fig. 7B).

### Conflict Analysis through the Prisms of Political Ecology and Game Theory

#### Unequal Distribution of Gains and (a)Symmetric ES Flows

In the news items analyzed, 41.5% of the relationships between stakeholders and WES in the Muga river basin were depicted as being positive/supportive. Most of the issues involved were related to the quantity of water and the management of water resources. The articles reported on high reservoir levels, the abundance of water in aquifers and wells, the connecting of villages and towns to the main water supply, the building of new wells to resolve water scarcity problems, and plans to build desalination plants to guarantee the supply of water for domestic use. The general interpretation was positive, but there was no discussion of how these decisions might affect different stakeholder groups or on how they were linked to water shortage problems.

These scenarios of mutual cooperation are fitting with the harmony game model and they coincided with a time of abundance, when all of the sectors, even the highly dependent urban-tourism, agricultural, and conservationist sectors, had free access to WES flows. This situation of harmony is reflected in some of the comments made during the interviews: *“There are no conflicts here. Sometimes you hear people saying that farmers water as much as they want, but there aren’t any conflicts. When there’s water and it rains a lot and the reservoir is full, we’re all happy. If water became scarce, then there would be competition. But not when we have water!” (interview# agricultural sector). “When there’s water, there’s water for everyone. For example, we haven’t complained that people are watering their gardens or filling their swimming pools because we’ve always had water. So why should I complain?” (interview# urban-tourism sector*). In short, when resources are plentiful, the stakeholders have no interest in entering into conflict with other stakeholders. Their actions and discourse reflect a spirit of cooperation. The power relationships are therefore symmetric. WES are perceived as a common pool resource, where there is rivalry (because the use of a resource by one party reduces its availability to another), but not excludability (because in a context of abundance, access to WES flows is equitable) (Nazari et al., 2020). In addition, the news items referred more frequently to bundles than trade-offs, indicating the existence of a management system that did not prioritize any one WES category (provisioning, regulating, cultural) over another (Dinar et al., 1992; Hicks et al., 2013).

The cross-sectional design of our study prevents us from drawing any conclusions regarding stakeholder behavior in the face of water scarcity, but it can be inferred from the comments made in the interviews that certain stakeholders might feel threatened than others and act accordingly.

Interview content analysis of stakeholder perceptions of water use and influence in the Muga river basin (Supplementary information C) showed that the group considered to use the most water (by 74.1% of the stakeholders interviewed) was the agricultural sector, followed by the tourism (63%) and urban (59%) sectors. These three groups were also considered to have a strong influence on decisions made regarding use of water resources (Supplementary information C). The conservationist sector was perceived as having a low-moderate level of influence. In line with the findings of Nash et al. (2013) and Lucci et al. (2014), the agricultural, tourism, and urban sectors can be “active and influential” agents, that is, agents capable of modifying the balance of ES flows.

In answer to the question *"Are you worried about less water availability in the river basin area and, if so, what do you think are the main causes?"* (Supplementary information B), 25 of the 27 stakeholders interviewed reported serious concerns about depleting water supplies and growing competition between new uses and new stakeholders. *“Yes, there’s a lot of competition, a lot, it’s very high. We’re all competing for the same resource and it’s becoming scarcer and scarcer. And it’s not just my opinion, that’s how it is, it’s a fact!”* (interview# agricultural sector). Climate change is one of the most widely perceived problems among the stakeholders in the Muga river basin, and this was increasingly reflected in the news coverage over the years. Mentions of the effects of climate change were particularly common in items on extreme weather events, such as floods, heavy rain, strong winds, and prolonged periods of drought, all associated with agricultural losses. Although numerous studies have shown that the media tends to depict all kinds of catastrophic news in a negative light to catch readers’ attention (McLellan & Shackleton, 2019), there is no doubt that climate change effects, together with a loss of biodiversity and declining water quantity and quality, have been instrumental in feeding tensions between sectors. *“Another problem is that the Muga river basin is small, and when it rains, it rains more in the other part of the basin, not at the reservoir. Everyone living here depends on that. With climate change we don’t know what’s going to happen, we don’t know whether we’ll have water or not. And without water, you can’t survive* (interview# agricultural sector).

The media coverage during periods of water scarcity revealed the existence of negative/conflictive relationships between stakeholders and between stakeholders and WES, denoting different degrees of perceived threat in relation to potential water shortages and suggesting a possible shift from an attitude of cooperation to one of competition. Competition typically increases in scenarios of shortage, as more influential groups may be able to impede or restrict access to certain WES flows by other groups (Felipe-Lucia et al., 2015; Fisher et al., 2014; Kull et al., 2015).

In total, 58.5% of the news items reported on conflictive or negative relationships in a scenario where WES changed from rival, non-excludable goods to rival, excludable goods. In line with previous reports (Costanza, 2008; Felipe-Lucia et al., 2015; Fisher et al., 2009), we found that provisioning WES (water for irrigation, domestic uses, and food production) were much more likely to be perceived as both rivalrous and excludable. On the contrary, certain regulating WES (water regulation and erosion control) and cultural WES (aesthetic values) WES tended to be perceived as non-rival, non-excludable goods.

Our findings also show that certain WES are what has been termed as “congestible” (Felipe-Lucia et al., 2015), meaning that they only cause conflict in certain situations. Examples of congestible WES in the current case study are biodiversity conservation, water regulation, water quality, and recreational activities, which changed from a non-rival to a rival, excludable good depending on their ecological status and relationship with the stakeholders who benefited from them (Table 2).

**Table 2:** Classification of WES according to rivalry, excludability, and congestibility based on analysis of media coverage of water resources in the Muga river basin between 2000 and 2020. Adapted from Felipe-Lucia et al. (2015). Red indicates rival goods (sources of conflict); yellow, congestible goods (potential sources of conflict depending on their status); and green, non-rival, non-excludable goods (not sources of conflict)

WES	Rival	Excludable	Congestible	Non-rival	Non-excludable
Irrigation water	**x**	**x**			
Domestic water	**x**	**x**			
Food production	**x**	**x**			
Biodiversity and ecosystem conservation			**x**	**x**	**x**
Water regulation			**x**	**x**	**x**
Water quality			**x**		
Aesthetic values				**x**	**x**
Recreational activities	**x**	**x**	**x**	**x**	
Cultural identity and educational values				**x**	**x**

**Fig. 8:**
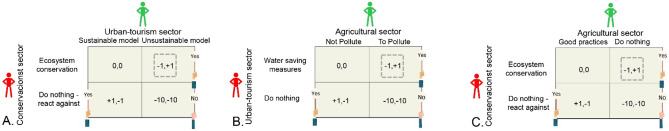
Scenario **A**. Conflict between conservationist and urban-tourism sectors, with the former blaming the latter for a growth and development model prioritizing the economy, with no consideration of the effects on biodiversity or water ecosystems. Scenario **B**. Conflict between urban-tourism sector and agricultural sector over groundwater contamination due to nitrate leakages and runoffs from crop and livestock farming and excessive extraction of water for agricultural purposes, also causing nitrate contamination and salinization of wells and placing pressure on water ecosystems; towns are frequently forced to advise against drinking tap water and to look for new water supply solutions, increasing pressure on the limited supply of water with an acceptable quality. Scenario **C**. Conflict between conservationist and agricultural sectors with the former blaming the latter for excessive water use related to inefficient irrigation techniques and the growing of water-intensive crops (corn and rice), with no consideration of ecosystems that receive insufficient flows to maintain an adequate ecological status. The gray square around the payoff indicates the real situation. Winners are shown in green and losers in red

Based on our news analysis, all the stakeholders employed a mixed strategy in the chicken game model. In other words, one of the parties chose to cooperate (with the goal of improving WES management) while the other chose not to (to maximize their gains from the WES without consideration of losses to the other party) (Fig. 8).

In the model shown in Fig. 8, built using data from the news coverage analysis, one of the stakeholders decides to do something to improve the distribution of WES flows with the aim of saving water and improving the sustainable management of water resources for the group as a whole, while the other decides to ignore these actions and continue as before (non-cooperative strategy). The numbers representing the payoffs come from theoretical models of game theory.

It has been shown that close cooperation is not always necessary to maximize gains, as stakeholders may sometimes cooperate and compete at the same time, depending on the situation and their short- or long-term vision of future gains (Najafi et al., 2013). This behavior has been defined as *coopetition* (Eleftheriadou & Mylopoulos, 2008).

As shown in the 2 × 2 games in Fig. 8, the urban-tourism and agricultural sectors emerged as winners at least once as they expected the other sector, not themselves, to do something to safeguard the WES. Again, the numbers representing the payoffs are from game theory models.

#### Stakeholder Opinions about the Water Situation in the River Basin

The observation that the urban-tourism and agricultural sectors emerged as winners in at least one situation is supported by some of the comments from the interviews: *“I think they take more water than others. Farmers still have a lot of work to do to save water, especially considering the type of crops they’re growing, which need a lot of water (e.g., corn and rice). We’re in an area where we shouldn’t have crops like these, we need more dryland crops, but these are less profitable, and as they are the ones moving more money, they get to decide” (interview# conservationist sector). “The perception that farmers have is that “if there was no tourism, I’d be able to water much more”. But the population exists so that’s not how it is, they also have to adapt to what others are doing. The urban consortium has to manage the water that comes through the Catalan Water Agency. As I’m from the urban sector, I’m not taking the water from you farmers, so I can do what I want” (interview # urban sector)*.

The conservationist sector came out losing in two of the conflict scenarios (Fig. 8A, C), positioning it as the least influential sector; this outcome is in line with the findings of the interviews, which showed that the conservationist sector adopts a cooperative strategy, even though it knows that the other sectors will not cooperate. *“We demand an increase in the minimum environmental flow because it’s disgraceful. We are in conflict with those in charge of managing and distributing water, but those in charge have the last word. There’s enough water for everyone, but it’s not distributed properly; and above all they don’t water properly, there are flooded roads, they water five fields instead of one. There’s a lot of collusion between sectors, the last thing on their mind is nature conservation, consumerism comes first, we know that's how things are, but we can’t do things differently. If we do, who’s going to protect the ecosystems and animals?”* (interview# conservationist sector).

Why does the conservationist sector choose to cooperate when it knows the other sectors will choose otherwise? As explained by Mulazzani et al. (2017), the existence of different groups of “active” stakeholders capable of directly modifying ES flows can give rise to a cause-effect relationship between behaviors. Our case study shows that a mixed strategy (with one stakeholder cooperating and the other not) led to a suboptimal outcome in each of the conflict scenarios. While this was not the best possible outcome for both parties, neither was it the worst based on the sum of gains (WES > 0). In brief, the two stakeholders reached the Nash equilibrium since they both needed to act in order to change and improve the benefits they could derive from the WES in the Muga river basin.

In a scenario of water scarcity, the stakeholders did not cooperate to achieve an equitable distribution of WES (WES = 0 for both parties). The interview pieces analyzed show how perceptions among stakeholders can be contradictory depending on their value systems. For instance, even if the conservationist sector decides to cooperate, its behavior may have no effect on the other stakeholders, who, depending on their value systems, interests, and level of influence, may even decide not to cooperate. This position is easier to understand through the prism of political ecology. Conservationists, for example, have a value system based on social behaviors and consensus that favors the common good and benefits ecosystems, biodiversity, and other stakeholders.

Analysis of conflicts through the prism of game theory revealed different strategies employed by the various stakeholder groups and provided insights into the preferences and value systems underlying their behaviors (Costanza et al., 2014; Fisher et al., 2014; Hicks et al., 2013). In our 2 × 2 game models, we did not observe any scenarios in which both parties chose not to cooperate, a strategy that would be detrimental to the basin’s water ecosystems and WES flows (Najafi et al., 2013).

In conclusion, in a resource-limited setting with restricted, unequal access to ES flows, identifying winners and losers in asymmetric power relationships provides valuable insights into who has the power to impede, control, or restrict access to natural resources, thereby generating fair or unfair trade-offs in the distribution of flows. Trade-offs can be affected by management decisions as well as by ecological status and stakeholder preferences and values (Bennett et al., 2009), and these decisions, in turn, can be influenced by the position and influence of different stakeholder groups.

#### Decisions and Fair and Unfair Trade-Offs

**Fig. 9:**
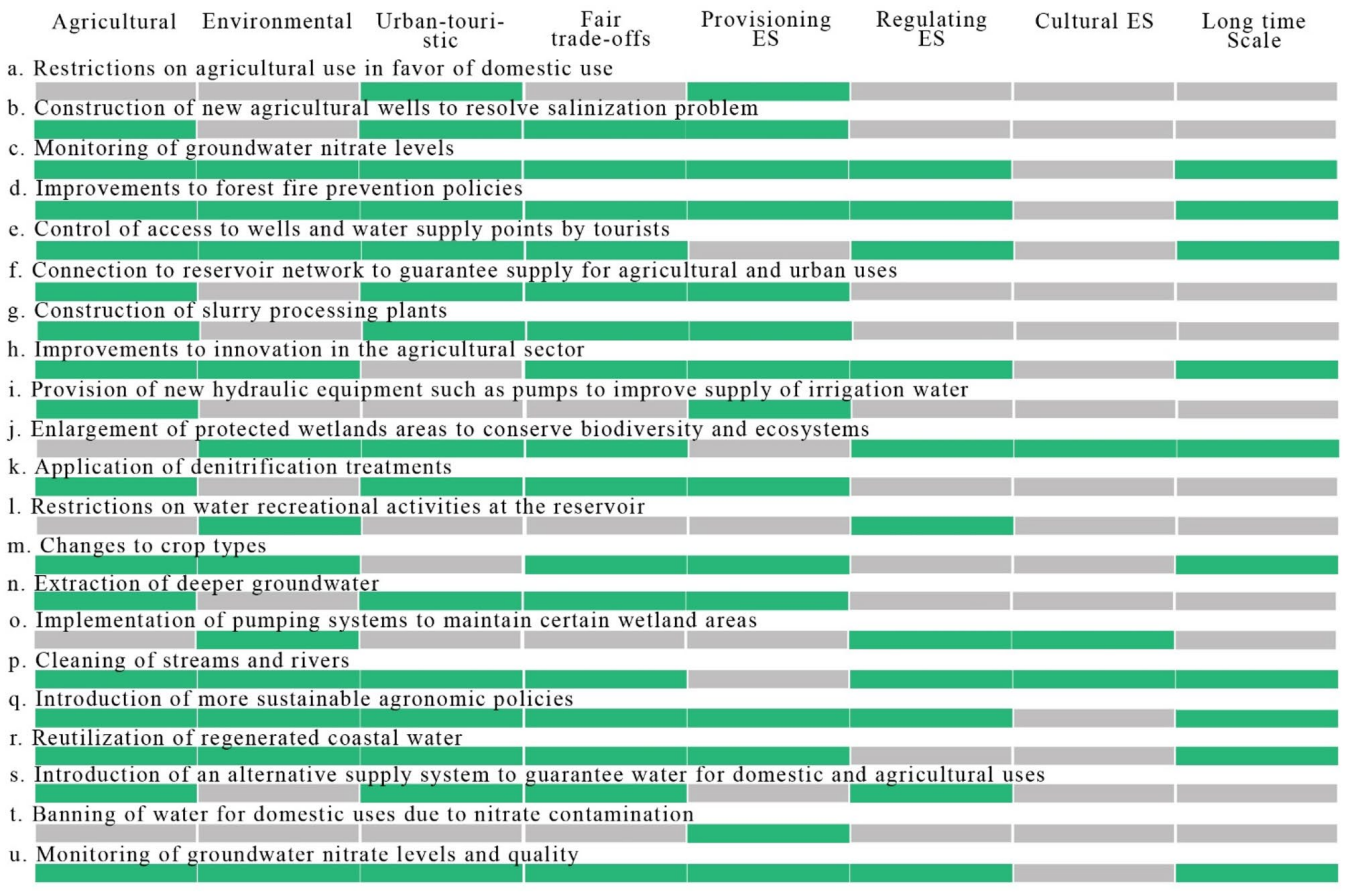
Analysis of the management solutions applied by the political-administrative sector described in the news between 2000 and 2020. The image summarizes the type of solution applied (letters a to u); the impact on stakeholder groups (green if the solution benefits a certain stakeholder group and gray if the solution provides no benefit); fair trade-offs (green if the solution benefits more than one stakeholder group, creating synergies and gray if the solution benefits just a one group with no thought given to others); synergies and trade-offs with WES categories (green if the solution generates an increase in a given WES category or synergies between categories and gray if the solution generates a decrease in a given WES category or trade-offs between categories). The last column, “long-term impact” indicates whether the solution reflected a long-term (green) or short-term vision (gray)

One of the aims of this study was to determine whether management decisions serving different interests of certain stakeholders groups can give rise to (in)justices (Forsyth, 2008) and alter WES flows (Lee, 2012; Mulazzani et al., 2017). Figure 9 summarizes the political measures taken to address WES-related problems described in the news and highlights the important role that politics and government can play in ensuring equity through control and access mechanisms. Government agencies, in theory, have the most power and are assumed to be neutral agents (Mulazzani et al., 2017) who take decisions aimed at maximizing environmental gains from ES flows and ensuring a fair distribution among multiple stakeholders. Their strategy should thus be a cooperative one, aimed at favoring win–win situations all round. Inevitably, however, top-down decisions and solutions will lead to trade-offs affecting both stakeholders and ES flows. Numerous studies have analyzed trade-offs to identify differential distributions in ES flows (Bennett et al., 2009; Castro et al., 2014; Cord et al., 2017; King et al., 2015; Kumar et al., 2011). In this study, we believed that such an analysis would not only show what happens when land management decisions increase the supply of a given ES to the detriment of another (King et al., 2015), but also uncover mechanisms of (in)justice underlying these decisions. Management decisions and policies affect the dynamics of entire socioecological systems, as they represent a social (legal/normative) consensus with the power to foster synergies between ES on the one hand and restrict use and access on the other (Felipe-Lucia et al., 2015; Podimata & Yannopoulos, 2015).

In our analysis of trade-offs resulting from political decisions taken between 2000 and 2020 (this period was based on the timeline in Fig. 5), we found that most decisions (15 of the 24 categories identified in the news analyzed) benefited more than one stakeholder group (Fig. 9). The remaining decisions, by contrast, benefited nobody or just a single group. This is the opposite of the Robin Hood philosophy, where the goal is to benefit as many people as possible (Fig. 9). We also saw that most of the solutions tended to increase the flow of provisioning WES creating trade-offs with regulating WES. One example of such a trade-off would be between irrigation water and greater ecological flows for environmental purposes. Analyses of this type can help identify whose interests ecosystem and WES management policies are designed to serve, that is, if their main intention is to increase provisioning ES, understood as economic, merchandisable goods (Kolinjivadi et al., 2020), such as water for irrigation, domestic use, and food production, or to regulate flows needed to safeguard water ecosystems. We found that many of the political decisions taken in the period analyzed were designed to increase the flow of provisioning WES (essentially water for irrigation and human consumption) with the aim of resolving problems in the agricultural and urban-tourism sectors. Much lower priority was given to regulating WES, such as water regulation, maintenance of habitats and aquatic biodiversity, and conservation.

Twelve of the government-level solutions were short-term solutions and included the construction of new or deeper wells and increased restrictions on the distribution of water between sectors. This tendency to provide stopgap solutions rather than tackle root causes highlights the lack of long-term vision. Analyzing solutions through the prism of game theory enables reflection on short- and long-term public sector goals and management priorities (ES categories), while a greater understanding of stakeholder preferences and potential modifiers of these preferences can help decision-makers take decisions based on shared and common values (Hicks et al., 2013) and implement fairer, more equitable land management policies that will benefit both ecosystems and future generations (Fig. 9).

## Conclusions

Application of a theoretical framework combining political ecology and game theory showed that WES conflicts can be deconstructed into their different dimensions. Our findings show that reflection on (a)symmetries in power relationships and decisions underlying human behavior is essential for understanding the value systems that define our priorities and relationships with others and with natural resources. Such a perspective is necessary if we are to transform asymmetric relationships into constructive ones and identify opportunities for an improved, more synergic use of “power”. The analytical framework employed enabled us to reflect on the impacts of human activities and decisions on ES flows. We found that power relationships in the basin are closely linked to concepts of control, access, equity, and justice. Each strategy is rooted in a value system that drives decisions on the use of natural resources and these decisions, in turn, have the power to generate trade-offs and situations of environmental (in)justice that lie at the root of these WES conflicts. In a scenario of limited access, the relationship between stakeholders may shift from one of cooperation to one of competition, reflecting the fragile nature of these relationships in the face of water scarcity and their strong dependence on the basin’s ecosystems. Consideration of WES as a common pool resource clearly highlights the need for an approach to natural resource management that is fair, inclusive, and effective.

In the current scenario of growing uncertainty and friction, depicted by the present case study, we are facing a new social tipping point, characterized by escalating climate change effects, ever-increasing conflicts over a scarce yet essential resource (water), and a need for strategies and actions that favor socioecological resilience and transformative, adaptive solutions. The COVID-19 pandemic is a clear example of this uncertainty and has revealed the extreme vulnerability of the prevailing economic and socioecological models. In the face of this new era of conflict, it is essential to identify and recognize power relationships to ensure that decisions are taken in pursuit of environmental justice at a multiscale level.

Further research is of course necessary to strengthen the methods employed in this study and validate their usefulness as an analytical framework for studying conflicts surrounding natural resources and examining interactions with social factors. Additional case studies in diverse applications and settings that differ both geographically and contextually will help deconstruct these conflicts into their different components and help truly capture the complexity of socioecological systems.

In conclusion, our analysis of WES conflicts reveals an aspect of environmental problems that is rarely taken into account: that these problems are not just ecological problems, but also “a symptom of dysfunctional societies and economies and impacted mainly on the poorest and most vulnerable people” (Forsyth, 2008:757). As such, they need to be viewed from an intellectual position of deconstructivist to break them down into their ecological, political, and sociocultural components. Only then will we gain a multidimensional understanding of the problems that exist and be able to take fairer and more equitable and sustainable decisions that will prioritize the common interests of present and future generations, both human and non-human.

## Supplementary Information

Below is the link to the electronic supplementary material.

Supplementary file1 (DOCX 113 KB)

## Data Availability

The author confirms that all data generated or analyzed during this study are included in this published article and its supplementary information files.
