# Supplementary material for: Deconstructing Ecosystem Service Conflicts through the Prisms of Political Ecology and Game Theory in a North-Western Mediterranean River Basin
Source: Hum Ecol Interdiscip J. 2022 May 26;50(3):477–92. doi: 10.1007/s10745-022-00325-5 (PMC9134714; doi:10.1007/s10745-022-00325-5)
Supplement: Supplementary file 1 — Supplementary file1 (DOCX 113 KB) [file 10745_2022_325_MOESM1_ESM.docx]

**Supplementary informations**

**Supplementary information A.** Interview model.

**Interview for the perception and mapping of ES by the stakeholders in the Muga river basin.**

(Explain the context of the research, the themes, the times, the modalities and the tools that will be used during the interview: the panel and the map)

**Date:**

**Interviewer:**

**Interviewed name:** …………………………………………………………….

**I. Familiarity of the interviewee with the study area**

- 1. Town or municipality in which you live …………………………………………………………
  2. Town or municipality in which you work …………………………………………………
  3. How many years have you lived in this landscape? ............................................................
  4. In your opinion, what are the limits of the Muga river basin? Please, draw the limits of the Muga river basin on the map. *(Give a short explanation of the study area, showing the map with the limits)*
  5. If you have to describe the Muga river basin to someone outside the basin, how would you describe it in a minute?

...........................................................................................................................................................................................................................................................................................................................................................................................................................................................................................................................................................................................................................................................................................................................................................................................................................................................................................................................................................................................................................................................................

1. **Level of knowledge and familiarity with the existence of ES**
   1. Do you think that this landscape, defined by the river basin and the different manifestations that the water element has in it, provides some type of benefit or positive effect for its well-being and the well-being of society?

A lot Enough

Few None

- 1. For example, which ones?

………………………………………………………………………………………………………………………………………………………………………………………………………………………………………………………………………………………………………………………………………………………………………………………………………………………………………

**2.3**. Have you ever heard the concept of Ecosystem Services? Yes No

¿and the concept of Environmental Services? Yes No

**2.4**. If yes, in what context?

....................................................................................................................................................................................................................................................................................................................................................................................................................................................................................................................................................................

*(Give a little explanation of the different SEs, showing the panel with the ES list)*

**III. Perception of the importance, demand and vulnerability of ES and identification and location of ES on the map.**

**3.1.** With the panel of different benefits (see ES Panel) provided by the watershed, could you say, in your opinion, which of these benefits are most important, and the degree of importance in the area?

Order the services using the numbers from 0 to 5, from the most important to the least important, and indicate the degree of importance of each one on a scale of 0 - 5.

(Order of importance: from the most to the least important; degree of importance: 0 = not perceived / not the case of the Muga; 1 = not important; 2 = not very important; 3 = important; 4 = very important; 5 = very important /essential)

| ES | | | | | | |
| --- | --- | --- | --- | --- | --- | --- |
| Order of importance | Degree of importance | | | | | |
|  | 0 | 1 | 2 | 3 | 4 | 5 |
|  |  |  |  |  |  |  |
|  |  |  |  |  |  |  |
|  |  |  |  |  |  |  |
|  |  |  |  |  |  |  |
|  |  |  |  |  |  |  |
|  |  |  |  |  |  |  |

*(Repeat the 3 questions 3.2.a, 3.2.b and 3.2.c for each ES and take a photo at the end of each map with the 3 layers)*

**MAP 3.2.a.** Of these benefits that you have valued as the most important, could you say which are the most important and strategic areas of the landscape for the production of these benefits? (Supply units, SPU, Service Provision Unit)

Put as many points as you want on the map, using these GREEN colour dots, trying to put dots for each ES.

(Show the map of the basin to help the interviewees identify and locate the areas on the map and place the dots of different colours, following the ES of the panel)

**MAPA 3.2.b.** Of these benefits that you have valued as the most important, could you say which are the most important and strategic areas of the landscape for the production of these benefits? (Benefiting Units, SBU, Service Benefiting Unit)

Put as many points as you want on the map, using these BLUE colour dots, trying to put dots for each ES.

(Show the map of the basin to help the interviewees identify and locate the areas on the map and place the dots of different colours, following the ES of the panel)

**MAPA 3.2.c.** Of these benefits that you have valued as the most important, could you say which are the most important and strategic areas of the landscape for the production of these benefits? (Vulnerable Units, dSPU, Service Vulnerable Unit)

Put as many points as you want on the map, using these ORANGE colour dots, trying to put dots for each ES.

(Show the map of the basin to help the interviewees identify and locate the areas on the map and place the dots of different colours, following the ES of the panel)

1. **Future options based on the influence of the driving forces of change and the perception of problems and concerns of the stakeholders.**

**4.1.** ¿Are you worried about a less water availability in the Muga river basin?

Yes No

**4.2.** What are the most serious water-related problems in the Muga river basin for you? Could you indicate the most important ones?

………………………………………………………………………………………………………………………………………………………………………………………………………………………………………………………………………………………………………………………………………………………………………………………………………………………………………………………………………………………………………………………………………………………………………………………………………………………………………………………………………………………………………………………………………………………………………………………………………………………………………………………………………………………

**4.3.** Of the problems that you have highlighted before, which sectors are most responsible for these water-related problems? Indicate the responsibility of each of the sectors, according to the high - medium - low level of responsibility.

|  | LEVEL OF WATER USE AND CONSUMPTION | | |
| --- | --- | --- | --- |
| Sector | High | Medium | Low |
| Urban |  |  |  |
| Agriculture |  |  |  |
| Tourism |  |  |  |
| Conservationist |  |  |  |
| Hydroelectric |  |  |  |
| Forest sector |  |  |  |
| Administrative |  |  |  |

**4.4.** Of the problems that you have highlighted before, which sectors do you think have the most power and influence in decision-making related to water management? Sort by level of influence high - medium - low.

|  | LEVEL OF INFLUENCE IN DECISION MAKING | | |
| --- | --- | --- | --- |
| Sector | High | Medium | Low |
| Urban |  |  |  |
| Agriculture |  |  |  |
| Tourism |  |  |  |
| Conservationist |  |  |  |
| Hydroelectric |  |  |  |
| Forest sector |  |  |  |
| Administrative |  |  |  |

**4.5.** If you had to distribute the available water in the Muga river basin using 100 points, could you distribute these 100 points according to who you think uses water and in what percentage?

| Sector | Points in % |
| --- | --- |
| Urban |  |
| Agriculture |  |
| Tourism |  |
| Conservationist |  |
| Hydroelectric |  |
| Forest sector |  |
| Administrative |  |

**4.6.** Do you think there is competition for the use of water resources? If this is the case, do you think that this competition is or could be an incentive or a barrier when implementing water saving measures? Can you justify your answers, please?

…………………………………………………………………………………………………………………………………………………………………………………………………………………………………………………………………………………………………………………………………………………………………………………………………………………………………………………………………………………………………………………………………………………………………………………………………………………………………………………………………………………………………………………………………………………………………………………………………………………………………………………………………………………………………………………………………………………………………………………………………………………………………………

**4.7** Do you think that working with ecosystem services for the well-being of people and society can help efficient water management in the basin?

Yes No I don’t know

**4.8.** Can you justify your answer, please?

……………………………………………………………………………………………………………………………………………………………………………………………………………………………………………………………………………………………………………………………………………………………………………………………………………………………………………………………………………………………………………………………………………………………………………………………………………………

**0. Socioeconomic profile of the interviewee**

- 1. Profession ……………………………………………………………….
  2. Place of birth …………………………………………………………………………….

0.3. Level of Studies:

- No studies
- ESO
- High School
- Vocational training
- University
  1. Age:
  2. Gender: Male Female Others I do not want to answer
  3. Net income level::

Less than 1000 Eur/month 2000-3000 Eur/month

1000-1500 Eur/month More than 3000 Eur/month

1500-2000 Eur/month

Complete by the interviewer:

- **Interview number:**
- **Date:**
- **Place:** …………………………………………………………………
- **Attitude of the interviewee:** active / indifferent / little engagement
- **Comprehension of the survey:** high / medium / low
- **Duration of the interview:**
- **Observations:**

**Supplementary information B.** Coding of the interview responses and categories derived from the content analysis (Code structure - categories - code frequency).

| Q4.2 What are the most serious water-related problems in the Muga river basin for you? Could you indicate the most important ones? |  |
| --- | --- |
| q4.2\Ignorance of the subject | 1 |
| q4.2\Territory and landscapes management | 1 |
| q4.2\Human pressures | 6 |
| q4.2\Human pressures \Lifestyle changes | 2 |
| q4.2\Human pressures \Urban-tourist growth | 5 |
| q4.2\Human pressures \Agriculture | 6 |
| q4.2\Climate change |  |
| q4.2\Climate change\Biodiversity-habitat loss | 6 |
| q4.2\Climate change\Fires | 2 |
| q4.2\Climate change\Temperature | 4 |
| q4.2\Climate change\Drought | 6 |
| q4.2\Climate change\Rain regime changes-decrease water | 17 |

| Q4.6 Do you think there is competition for the use of water resources? If so, do you think that this competition is or could be an incentive or a barrier when implementing water saving measures? Can you justify your answers, please? |  |
| --- | --- |
| q4.6\Water management proposal | 3 |
| q4.6\No competition | 5 |
| q4.6\Barrier | 3 |
| q4.6\Incentive | 3 |
| q4.6\Yes-No competition (it depends on the time of the year or available water) | 4 |
| q4.6\Competition |  |
| q4.6\Competition\Urban sector | 2 |
| q4.6\Competition\Tourism sector | 1 |
| q4.6\Competition\Agricultural sector | 7 |
| q4.6\Competition\Agricultural sector\Agricultural sector- Environmental groups | 4 |
| q4.6\Competition\Agricultural sector\Agricultural sector-Administrative sector | 1 |
| q4.6\Competition\Agricultural sector\Agricultural sector-Urban sector | 3 |
| q4.6\Competition\Agricultural sector\Agricultural sector-Tourism sector | 6 |
| q4.6\Competition\Administrative sector- Environmental groups | 1 |
| q4.6\Competition\Tourism sector-Urban sector-Environmental groups | 3 |
| q4.6\Competition\Participatory processes | 2 |
| q4.6\Competition\Administrative sector | 8 |

**Supplementary information C.** Comparison between the level of water use and consumption by stakeholder sectors (A) and the level of influence in decision-making (B) according to stakeholder perceptions.

| (A) LEVEL OF WATER USE AND CONSUMPTION | | | | (B) LEVEL OF INFLUENCE IN DECISION MAKING | |
| --- | --- | --- | --- | --- | --- |
| SECTOR | LEVELS | COUNTS | % OF TOTAL | COUNTS | % OF TOTAL |
| Urban | High | 16 | 59.3 | 17 | 63.0 |
|  | Medium | 8 | 29.6 | 4 | 14.8 |
|  | Low | 3 | 11.1 | 6 | 22.2 |
|  | Does not have |  |  |  |  |
| Agriculture | High | 20 | 74.1 | 13 | 48.1 |
|  | Medium | 7 | 25.9 | 8 | 29.6 |
|  | Low |  |  | 5 | 18.5 |
|  | Does not have |  |  | 1 | 3.7 |
| Tourism | High | 17 | 63.0 | 17 | 63.0 |
|  | Medium | 7 | 25.9 | 3 | 11.1 |
|  | Low | 3 | 11.1 | 6 | 22.2 |
|  | Does not have |  |  | 1 | 3.7 |
| Conservationist | High |  |  | 3 | 11.1 |
|  | Medium | 8 | 63.0 | 10 | 37.0 |
|  | Low | 17 | 29.6 | 13 | 48.1 |
|  | Does not have | 2 | 7.4 | 1 | 3.7 |
| Hydroelectric | High | 3 | 11.1 | 3 | 11.1 |
|  | Medium | 7 | 25.9 | 5 | 18.5 |
|  | Low | 13 | 48.1 | 16 | 59.3 |
|  | Does not have | 4 | 14.8 | 3 | 11.1 |
| Forest sector | High | 1 | 3.7 | 1 | 3.7 |
|  | Medium | 6 | 22.2 | 4 | 14.8 |
|  | Low | 17 | 63.0 | 20 | 74.1 |
|  | Does not have | 3 | 11.1 | 2 | 7.4 |
| Administrative | High |  |  | 25 | 92.6 |
|  | Medium |  |  | 2 | 7.4 |
|  | Low |  |  |  |  |

**Supplementary information D.** Codes and sub-codes used in the inductive coding process.

https://github.com/enrigarau/Deconstructing-ecosystem-service-conflicts-through-the-prisms-of-political-ecology-and-game-theory.git
